# Fatal *Babesia canis canis* infection in a splenectomized Estonian dog

**DOI:** 10.1186/s13028-016-0189-4

**Published:** 2016-01-25

**Authors:** Valentina Tiškina, Valentina Capligina, Külli Must, Inese Berzina, Renate Ranka, Pikka Jokelainen

**Affiliations:** 1 Estonian University of Life Sciences, Kreutzwaldi 62, 51014 Tartu, Estonia; 2 Latvian Biomedical Research and Study Centre, Ratsupites Street 1, Riga, 1067 Latvia; 3 Faculty of Veterinary Medicine, Latvia University of Agriculture, Helmana Street 8, Jelgava, 3004 Latvia; 4 Riga Stradiņš University, Dzirciema Street 16, Riga, 1007 Latvia; 5 University of Helsinki, P.O. Box 66, 00014 Helsinki, Finland

**Keywords:** Blood smear, Canine babesiosis, Europe, Splenectomy

## Abstract

A previously splenectomized dog from Estonia was presented with a sudden lack of appetite and discoloration of the urine. Despite supportive therapy, its condition deteriorated dramatically during 1 day. Severe thrombocytopenia and high numbers of protozoan hemoparasites were evident in blood smears, and the hematocrit dropped from 46 to 33 %. The dog was euthanized before specific antibabesial treatment was initiated. Blood samples from the dog and from two other dogs in the same household tested positive for *Babesia* using molecular methods, and the sequences of partial 18S rRNA gene confirmed the causative species as *Babesia canis canis*. The risk of severe, rapidly progressing babesiosis in splenectomized dogs merits awareness.

## Background

Canine babesiosis is a hemoprotozoan disease, which has been diagnosed in increasing numbers in north-eastern Europe in the past decade [1–3]. The clinical signs occur 1–2 weeks after infection and include inappetence, fever, anemia, pigmenturia, thrombocytopenia, and—if the patient has a spleen—splenomegaly [4, 5]. In both human and canine patients, splenectomy is a major risk factor for a severe course of infection with *Babesia* spp. [4–7]. Here we report an acute and rapidly fatal case of babesiosis in a splenectomized dog in Estonia.

## Case presentation

In March 2015, a 4.5-year-old male borzoi (dog 1) was brought to the small animal clinic of the Estonian University of Life Sciences. The main complaints were lethargy and change in urine color, which the owner had noted the same morning. Lack of appetite had been evident since the previous evening, i.e., for less than 12 h.

The dog was a show and breeding dog, routinely vaccinated and treated with anthelmintics, and apparently healthy. In 2013, the dog had a surgical intervention for gastric dilatation and splenic flexure where gastropexy and splenectomy were performed.

The dog had attended a show in the United Kingdom 13 days before being admitted to the clinic. The dog had not received prophylactic treatment against ticks prior to the travel to the United Kingdom, which was done via Poland, Germany, and France. The owner did not observe ticks on the dog.

The dog was lethargic at clinical examination and had signs of pain in the caudal abdomen. It had pale mucous membranes, and the capillary refill time was >2 s. Its heart rate was 130 beats per min, while the respiratory rate was 20 per min. Rectal temperature was 39.4 °C. Diagnostic radiographic imaging of the thorax and abdomen were unremarkable. Because free catch urine was dark brown, the refractometer could not define its special gravity and urine dipstick analysis results were unreadable. Microscopic examination of the urine sample revealed numerous erythrocytes and some coccus-shaped bacteria, which were considered a contamination and not identified further.

**Fig. 1:**
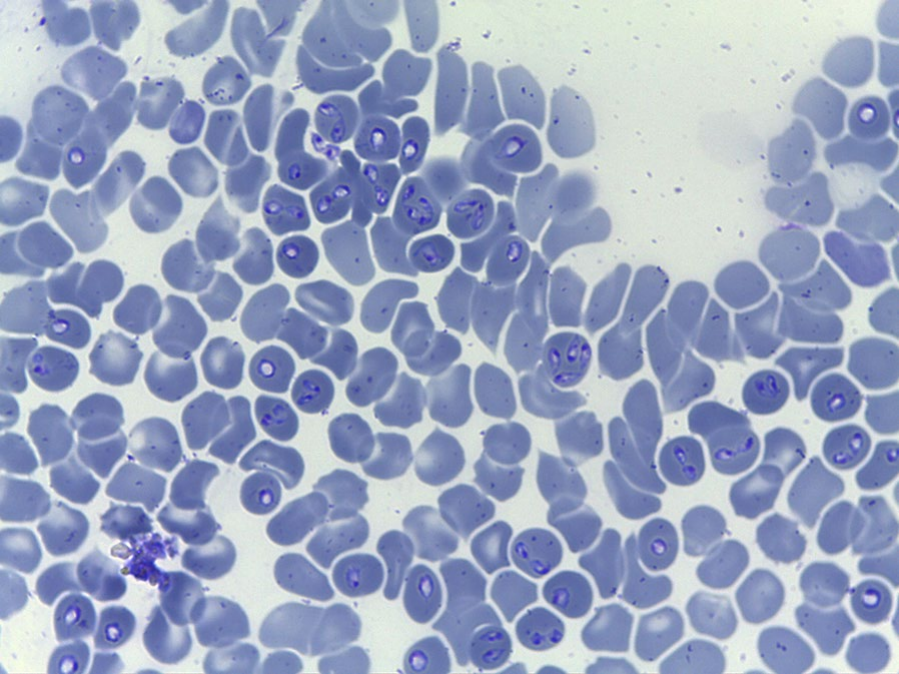
Numerous intraerythrocytic parasites, later confirmed as *Babesia canis canis*, in the blood smear of dog 1. May-Grünwald-Giemsa stain

Blood samples for hematology and biochemical analysis were collected from peripheral vein about 1 h after admittance. The complete blood count (Celltac α type MEK-6400 K, Nihon Kohden, Japan) (Table 1) and blood smears stained with May-Grünwald-Giemsa showed severe thrombocytopenia and lymphocytopenia, but no anemia. Blood smears revealed a massive infection of erythrocytes with tear-shaped protozoan hemoparasites (Fig. 1). The results of serum biochemical analysis (IDEXX VETTEST^®^ 8008, Tokyo, Japan) (Table 2) showed elevated aspartate aminotransferase, total bilirubin, and urea values. *Leptospira* spp. quick test (FASTest^®^ LEPTOSPIRA IgM ad us. vet. Diagnostik MEGACOR, Hörbranz, Austria) was negative.

**Table 1:** Hematological findings of dog 1

Parameter	Result	Reference interval
Erythrocytes (/l)	6.33 × 10^12^	5.5–8.5 × 10^12^
Hemoglobin concentration (g/l)	157	120–180
Hematocrit (%)	45.7	37–55
Mean corpuscular volume (fl)	72.2	64–79
Mean corpuscular hemoglobin concentration (g/l)	344	300–350
Leukocytes (*/*l)	*5.3 × 10* ^*9*^	6–12 × 10^9^
Segmented neutrophils (/l)	4.8 × 10^9^	4.3–7.6 × 10^9^
Band neutrophils (/l)	0.0 × 10^9^	0–0.3 × 10^9^
Lymphocytes (/l)	*0.3 × 10* ^*9*^	1.1–3.0 × 10^9^
Eosinophils (/l)	*0.0 × 10* ^*9*^	0.2–0.8 × 10^9^
Monocytes (/l)	0.2 × 10^9^	0.2–0.7 × 10^9^
Basophils (/l)	0.0 × 10^9^	0–0.2 × 10^9^
Platelets (/l)	*45 × 10* ^*9*^	150–500 × 10^9^

Values outside the reference interval are high-lighted in italics

**Table 2:** Serum biochemical analysis of dog 1

Parameter	Result	Reference interval
Alanine aminotransferase (U/l)	57	10–100
Aspartate aminotransferase (U/l)	*230*	0–50
Alkaline phosphatase (U/l)	68	23–212
Creatinine (µmol/l)	146	44–159
Urea (mmol/l)	*10.7*	2.5–9.6
Total bilirubin (µmol/l)	*80*	0–15
Potassium (mmol/l)	3.8	3.5–5.8
Sodium (mmol/l)	146	144–160
Chloride (mmol/l)	113	109–122

Values outside the reference interval are high-lighted in italics

Mildly elevated urea and a total bilirubin value of 80 µmol/l (reference interval 0–15 µmol/l) suggested that anemia could have been present but masked. Although dehydration was not noted in the physical examination, it was suspected, as the dog had had a lack of appetite for 12 h.

Supportive treatment included fluid therapy (Ringer lactate 2 ml/kg/h) and analgesia (buprenorphine 0.01 mg/kg q6h). Specific treatment with imidocarb dipropionate was not available at the clinic and was ordered and expected to arrive the same day. The dog’s condition deteriorated rapidly. Within 8 h after admission it developed epistaxis, and soon after it went into unresponsive seizures. At the time of seizures, the dog’s rectal temperature was 40.3 °C, and the hematocrit had dropped to 33 %. The dog was euthanized, with the owner’s consent, before the specific treatment started. Necropsy was not performed.

On the same day, blood samples were tested from two other dogs (dogs 2 and 3) that belonged to the same owner and were among the dogs that traveled to the United Kingdom for the dog show. The complete blood count of dog 2 revealed leukopenia (3.8 × 10^9^/l) and lymphocytopenia (0.5 × 10^9^/l). Dog 3 had lymphocytopenia (0.9 × 10^9^/l) and anisocytosis. Lymphocytopenia is not an unusual hematological finding in canine, and human, babesiosis [8, 9]. The blood smear of dog 2 showed few erythrocytes infected with parasites, whereas none were seen in the blood smear of dog 3. Both dogs tested negative for antibodies against *Babesia* with a commercial test (Laboklin, Bad Kissingen, Germany). Dog 2 had another positive blood smear 3 days later, and showed mild lethargy and lack of appetite. It was administered two doses (6.6 mg/kg) of imidocarb dipropionate subcutaneously with a 14 days interval, and remained clinically stable. Dog 3 had recently received a course of chemotherapy for osteosarcoma, and the owner refused its anti-babesial treatment.

An ethylenediaminetetraacetic acid (EDTA) anticoagulated whole blood sample from each dog was tested with polymerase chain reaction (PCR) for *Babesia* sp. For DNA isolation, 1 ml of blood was lysed with three volumes of lysis buffer (0.32 M sucrose, 10 mM Tris–HCl (pH 7.6), 5 mM MgCl_2_, 1 % Triton-X-100) for 15 min at 4 °C and centrifuged at 3220*g* for 15 min at 4 °C. The pellet was washed with 5 ml of cell suspension solution (25 mM EDTA [pH 8.0], 200 mM NaCl) and centrifuged at 3220*g* for 10 min at 4 °C. The pellet was dissolved in 2.5 ml of cell suspension solution to which 200 μl of 10 % sodium dodecyl sulfate and 2.5 μl of proteinase K (600 U/ml, Thermo Scientific, Waltham, USA) were added for cell digestion for 1 h at 50 °C. DNA extraction was performed with an equal volume of buffered phenol (pH 8.0) and then an equal volume of chloroform. The DNA was precipitated with an equal volume of isopropanol and collected by centrifugation at 3220*g* for 10 min at 4 °C. The pellet was washed with ice-cold 70 % ethanol, dried, and dissolved in 250 µl of 1 × TE buffer (10 mM Tris–HCl, 1 mM EDTA [pH 8.0]). DNA concentration and purity were measured in a spectrophotometer (ND-1000 UV–VIS Spectrophotometer, NanoDrop Technologies, Wilmington, USA).

*Babesia* sp. specific DNA was detected by amplification of the 18S rRNA gene fragment [10]. PCRs were performed in a thermocycler (Mastercycler epgradientS, Eppendorf, Hamburg, Germany) in a final volume of 25 μl, containing 1 × Phusion Buffer (with MgCl_2_), 200 µM of each dNTPs, 0.3 µM of each primer (5–22F was used as forward primer and 1661R as reverse primer [10]), 0.4 U of Phusion Hot Start II DNA Polymerase, and 1 µl of DNA. Cycling conditions were initial denaturation at 98 °C for 30 s, followed by 40 amplification cycles (98 °C for 10 s, 65 °C for 30 s, and 72 °C for 30 s), and a final extension step at 72 °C for 5 min. DNA from *B. canis canis* positive dog blood (Lv-dog 2, GenBank Nr. JX227980, [1]) served as the positive control, and PCR mix without DNA as the negative control. PCR products of approximately 2000 base pairs were visualized by electrophoresis in a 1.5 % agarose gel (TopVision Agarose, Thermo Scientific, Waltham, USA) in 1 × Tris–Acetate-EDTA buffer containing 0.2 µg/ml ethidium bromide with UV light. Primers were synthetized by Metabion International AG (Steinkirchen, Germany), and all PCR reagents were obtained from Thermo Scientific (Waltham, USA).

The positive PCR products were subjected to sequencing using the BigDye Terminator v3.1 Cycle Sequencing Kit (Applied Biosystems, Carlsbad, USA) according to the manufacturer’s standard protocol. For better coverage for the sequence of the 18S rRNA gene, PCR products were sequenced with four primers: 5–22 F and 455–479 F were used for the sense chain, and 1661R and 793–772R were used for the antisense chain [10]. The sequenced material was analyzed with an ABI Prism 3100 Genetic Analyzer (Perkin-Elmer, Waltham, USA). All sequence chromatograms were viewed and edited using Finch TV Version 1.4.0 software (Geospiza Inc., Seattle, WA, USA). Primer sequences were omitted in all sample sequences. Sequences generated in the present study were compared with DNA sequences deposited in the NCBI GenBank database [11] using the BLAST program [12, 13].

**Fig. 2:**
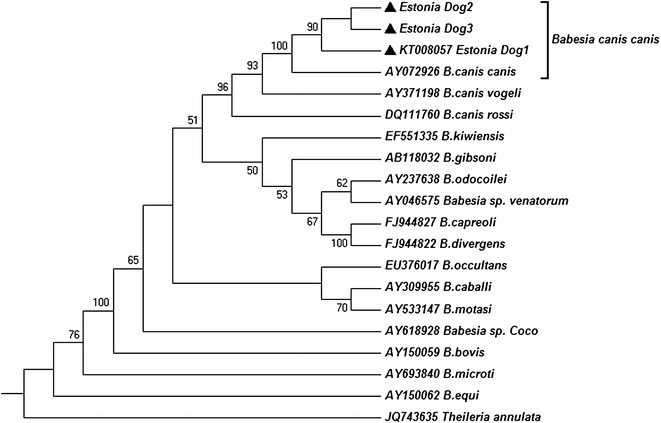
The phylogenetic relationship of *Babesia* species, including the *Babesia canis canis* from dogs 1–3 (*filled triangles*) of the present study

**Fig. 3:**
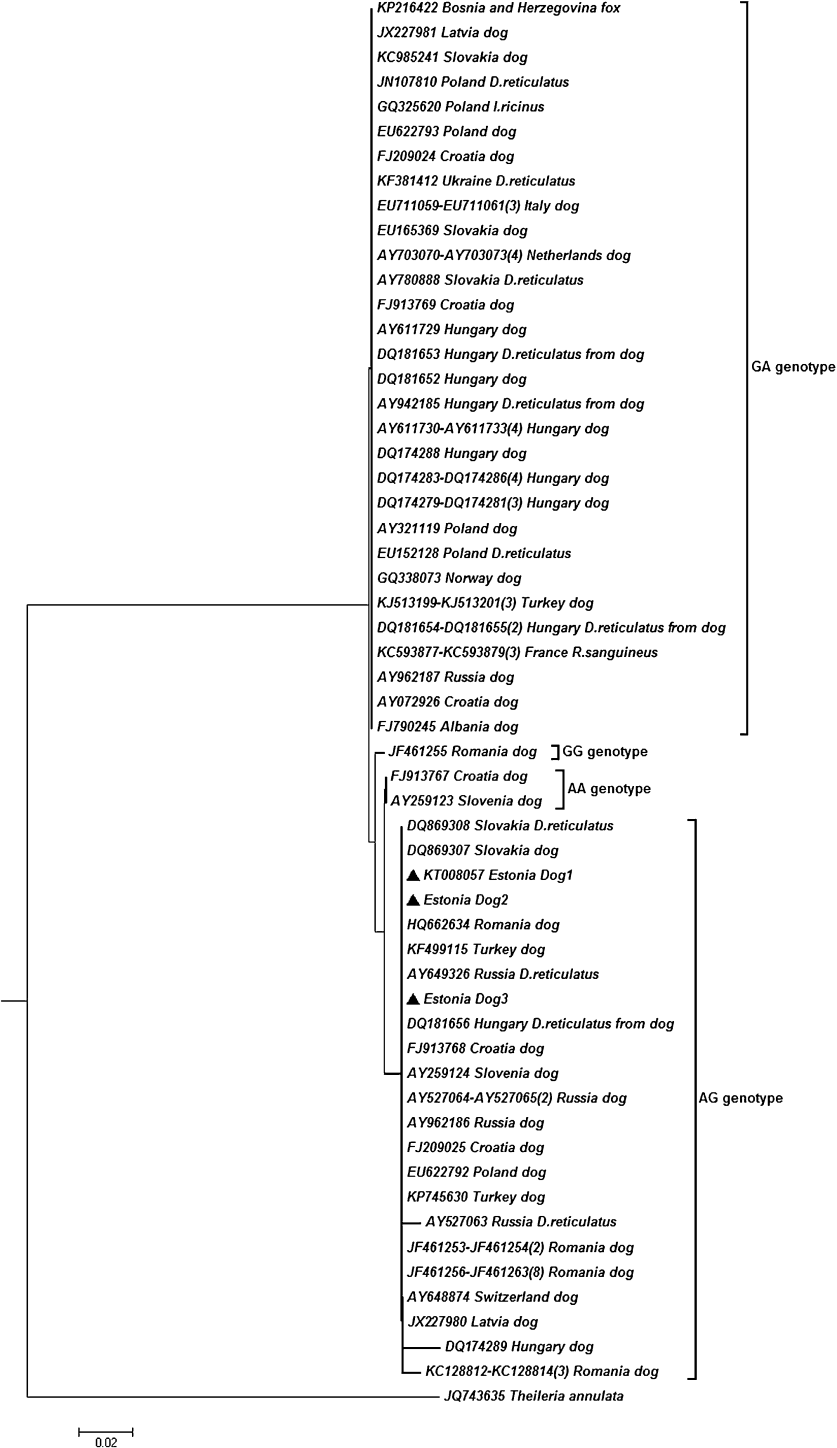
Phylogenetic relationship tree of *Babesia canis canis*, including the sequences identified in the present study (*filled triangles*). Taxa name consists of the GenBank accession number, country of origin, and a host of origin; a digit in parenthesis indicates the number of identical sequences. *D. reticulatus, Dermacentor reticulatus*; *I. ricinus, Ixodes ricinus*; *R. sanguineus*, *Rhipicephalus sanguineus*

Phylogenetic relationships between the *B. canis canis* from dogs 1–3 and other *Babesia* spp. from the NCBI GenBank database were established using the neighbor-joining method. Phylogenetic analyses were conducted in MEGA6 software version 6.0 package [14]. For the constructing of the neighbor-joining phylogenetic tree (Figs. 2, 3), the evolutionary distances were calculated according to the Jukes-Cantor parameter model and are in the units of the number of base substitutions per site. Jukes-Cantor DNA substitution model was selected according to Maximum Likelihood analysis of 24 different nucleotide substitution models conducted on MEGA6 due to the lowest BIC scores (Bayesian Information Criterion). *Theileria annulata* was used as an out-group. The included codon positions were 1st + 2nd + 3rd + noncoding. All positions containing gaps and missing data were eliminated from the dataset (complete deletion option). The rate variation among sites was modeled with a gamma distribution (shape parameter = 1). The robustness of the generated phylogenetic tree was evaluated by bootstrap analysis of the original tree by 500 replicates. The percentage of replicate trees in which the associated taxa clustered together in the bootstrap test is shown next to the branches in Figs. 2 and 3 (only values above 50 displayed). The scale bar represents the units of the number of base substitutions per site. Vertical lengths are not significant and were set only for clarity.

The blood samples of all three dogs were PCR-positive. The sequences from dogs 1–3 were identical, and genetically similar to *B. canis canis* sequences in GenBank database (Fig. 2). The *B. canis canis* 18S rRNA sequence from dog 1 was deposited in the GenBank database under the accession number KT008057.

A further phylogenetic analysis of partial 18S rRNA gene sequences was conducted (Fig. 3), using neighbor-joining methods and *T. annulata* as an out-group. The robustness of the generated phylogenetic tree of *B. canis canis* was evaluated by bootstrap analysis of the original tree by 500 replicates (the percentage of replicate trees in which the associated taxa clustered together in the bootstrap test are not shown, was lower than 50). Evolutionary analyses were conducted in MEGA6 [14]. The phylogenetic analysis showed that the sequences from this study were identical to other European *B. canis canis* isolates that display AG genotype at positions 610/611 of the whole-length gene [15, 16].

## Conclusion

The infections were caused by *B. canis canis*. Based on the time between traveling in the United Kingdom, Poland, Germany, and France and the onset of clinical signs of babesiosis in dog 1, and the lack of antibodies against *Babesia* in dogs 2 and 3, the described infections were likely related to the travel. However, autochthonous *B. canis* infections have been diagnosed in the other Baltic countries than Estonia [1, 17] and it is therefore possible that the infections were acquired in Estonia.

Based on the increasing number of cases diagnosed, babesiosis should be included in the list of differential diagnoses for dogs with inappetence, fever, anemia, and pigmenturia [4, 5], also in north-eastern Europe. Dog 1 had pale mucous membranes but no apparent decrease in the hematocrit at admission, and it was the evaluation of blood smears that allowed the early diagnosis. Our case report is a useful reminder of the high diagnostic importance of blood smears.

The course of the disease in dog 1 illustrates how acute babesiosis can progress rapidly towards a fatal outcome in splenectomized dogs. Splenectomy as iatrogenic risk merits attention from both preventive and clinical aspects. Preventing babesiosis could be emphasized, for example, in the post-surgery information given to animal owners. Splenectomized patients with suspected babesiosis should be considered emergency patients. It is noteworthy that the first clinical signs of babesiosis may be mild or unspecific, while it is important to reach or rule out the diagnosis without delay.
